# Diagnosis and treatment of anti‐insulin antibody‐mediated labile glycaemia in insulin‐treated diabetes

**DOI:** 10.1111/dme.15194

**Published:** 2023-09-01

**Authors:** David S. Church, Peter Barker, Keith A. Burling, Shah K. Shinwari, Carmel Kennedy, Diarmuid Smith, David P. Macfarlane, Andrew Kernohan, Anna Stears, Muhammad A. Karamat, Karen Whyte, Parth Narendran, David J. Halsall, Robert K. Semple

**Affiliations:** ^1^ Department of Clinical Biochemistry and Immunology Cambridge University Hospitals NHS Foundation Trust Cambridge UK; ^2^ The University of Cambridge MRC Metabolic Disease Unit Wellcome Trust‐MRC Institute of Metabolic Science Cambridge UK; ^3^ Core Biochemical Assay Laboratory NIHR Cambridge Biomedical Research Centre Cambridge UK; ^4^ Diabetes & Endocrinology Centre Birmingham Heartlands Hospital Birmingham UK; ^5^ Department of Diabetes and Endocrinology Beaumont Hospital, RCSI Medical School Dublin Dublin Ireland; ^6^ Department of Diabetes & Endocrinology Raigmore Hospital Inverness UK; ^7^ Department of Diabetes and Endocrinology Queen Elizabeth University Hospital Glasgow UK; ^8^ National Severe Insulin Resistance Service, Wolfson Diabetes & Endocrine Clinic Cambridge University Hospitals NHS Foundation Trust Cambridge UK; ^9^ West Glasgow Ambulatory Care Hospital Glasgow UK; ^10^ Institute of Metabolism and Systems Research, College of Medical and Dental Sciences University of Birmingham Edgbaston UK; ^11^ University of Edinburgh Centre for Cardiovascular Science Queen's Medical Research Institute Edinburgh UK

**Keywords:** anti‐insulin antibodies, diabetes mellitus, gel filtration chromatography, Hirata disease, immunoassay, insulin autoimmune syndrome, polyethylene glycol

## Abstract

**Aims:**

Anti‐insulin antibodies in insulin‐treated diabetes can derange glycaemia, but are under‐recognised. Detection of significant antibodies is complicated by antigenically distinct insulin analogues. We evaluated a pragmatic biochemical approach to identifying actionable antibodies, and assessed its utility in therapeutic decision making.

**Methods:**

Forty people with insulin‐treated diabetes and combinations of insulin resistance, nocturnal/matutinal hypoglycaemia, and unexplained ketoacidosis were studied using broad‐specificity insulin immunoassays, polyethylene glycol (PEG) precipitation and gel filtration chromatography (GFC) with or without ex vivo insulin preincubation.

**Results:**

Twenty‐seven people had insulin immunoreactivity (IIR) below 3000 pmol/L that fell less than 50% after PEG precipitation. Insulin binding by antibodies in this group was low and judged insignificant. In 8 people IIR was above 3000 pmol/L and fell by more than 50% after PEG precipitation. GFC demonstrated substantial high molecular weight (HMW) IIR in 7 of these 8. In this group antibodies were judged likely significant. In 2 people immunosuppression was introduced, with a good clinical result in one but only a biochemical response in another. In 6 people adjustment of insulin delivery was subsequently informed by knowledge of underlying antibody. In a final group of 5 participants IIR was below 3000 pmol/L but fell by more than 50% after PEG precipitation. In 4 of these GFC demonstrated low levels of HMW IIR and antibody significance was judged indeterminate.

**Conclusions:**

Anti‐insulin antibodies should be considered in insulin‐treated diabetes with unexplained glycaemic lability. Combining immunoassays with PEG precipitation can stratify their significance. Antibody depletion may be beneficial, but conservative measures often suffice.

**Novelty statement**

**What is already known?**

Anti‐insulin antibodies can cause dysglycaemia in exogenous insulin‐naïve people (‘insulin autoimmune syndrome’) and in people with insulin‐treated diabetes. Metabolically beneficial depletion of insulin‐binding antibodies has been reported.

**What this study has found?**

Using a standardised laboratory assessment in 40 insulin‐treated participants clinically suspected of having anti‐insulin antibody‐mediated dysglycaemia, the condition was excluded in 29 individuals. 7 people were identified with highly likely clinically significant antibodies for which immunomodulation was considered, and 4 people exhibited insulin binding of possible clinical significance.

**What are the implications of the study?**

Identification of anti‐insulin antibody‐mediated dysglycaemia in insulin‐treated participants is challenging, however, may explain glycaemic lability and guide immunomodulation in severe cases.

## 1 INTRODUCTION

Anti‐insulin antibodies are common in insulin‐treated diabetes^1^ and/or autoimmune insulitis,^2^, ^3^, ^4^ however, most do not produce clinically significant derangement of insulin kinetics.^5^, ^6^, ^7^ It has long been recognised, however, that at sufficient concentration and affinity, anti‐insulin antibodies delay insulin clearance from the blood.^1^, ^8^, ^9^ This can produce insulin resistant hyperglycaemia^10^, ^11^, ^12^, ^13^ and/or ketoacidosis^14^, ^15^ at one extreme, prolonged hypoglycaemia^10^, ^14^, ^15^, ^16^, ^17^, ^18^ at the other, and sometimes both. Clearance of bioactive insulin from antibody‐bound reservoirs may be sufficiently retarded to produce sustained hypoglycaemia for days following cessation of exogenous insulin even in those with endogenous insulin deficiency.^18^, ^19^ For some people with insulin‐treated diabetes and anti‐insulin antibody‐mediated dysglycaemia, antibody depletion therapy has been demonstrated to have clinical benefit.^10^, ^13^, ^16^, ^17^

Anti‐insulin antibody‐mediated dysglycaemia in insulin‐treated diabetes was intensively studied in the era of widespread animal insulin use,^20^ and to some extent later when insulin analogues came to predominate.^5^, ^6^, ^7^ It has also been well studied in exogenous insulin‐naïve participants, in whom we have used combinations of insulin immunoassays, polyethylene glycol (PEG) precipitation and gel filtration chromatography (GFC) to identify disease warranting antibody depletion.^21^, ^22^, ^23^ It has not been addressed in a similarly concerted way in insulin‐treated diabetes since genetically modified insulin analogues became widely used, although each of these analogues is antigenically distinct from native insulin. Structurally distinct insulin analogues, however, pose an analytic challenge, as immunoassays show widely variable ability to detect different analogues.^24^, ^25^ Resulting uncertainty around optimal biochemical investigation of people with suspected anti‐insulin antibodies who may benefit from immunodepletion is a major barrier to optimal management. This is of particular importance as dysglycaemia, particularly prolonged episodic hypoglycaemia, can masquerade as wilful insulin dose manipulation, sometimes with forensic or safeguarding implications.

Drawing on our recent experience in exogenous insulin‐naïve participants with insulin autoimmune syndrome (IAS, Hirata disease),^21^, ^22^, ^23^ we undertook standardised laboratory assessment of participants with insulin‐treated diabetes and labile glycaemic control in whom anti‐insulin antibodies were clinically suspected. We present investigation of 40 participants, using published methods^21^ to assess prevalence of actionable anti‐insulin antibodies. We identified 7 participants with anti‐insulin antibody‐mediated labile diabetes who were potential candidates for immunosuppressive therapy, and a further 5 participants with a lesser degree of antibody‐bound insulin of possible clinical significance.

## 2 METHODS

### 2.1 Participants

All studies were performed in accordance with the Declaration of Helsinki (2008). Insulin‐treated people with diabetes were evaluated by the UK Severe Insulin Resistance Supraregional Assay Service, Cambridge University Hospitals, Cambridge over a 9 year period. In all participants the possibility of significant anti‐insulin antibodies had been raised due to labile glycaemia, defined by different combinations of unexplained exogenous insulin resistance (high or rapidly increasing subcutaneous and/or intravenous insulin requirement), unexplained daytime hyperglycaemia with nocturnal/matutinal hypoglycaemia, and/or unexplained recurrent diabetic ketoacidosis. 40 individuals with a complete dataset were included in the study. Non‐fasting blood was collected in heparin‐ or gel‐containing tubes, and placed directly on ice or coagulated at room temperature before storage on ice for plasma and serum separation, respectively. After centrifugation plasma/serum was frozen at −80°C until analysis. Samples were taken at presentation and serially to monitor treatment response where indicated.

### 2.2 Biochemical studies

Serum anti‐insulin IgG concentration was measured using an in‐house human insulin specific ELISA incorporating ImmunoCAP™ reagents (Thermo Fisher Scientific). Plasma insulin immunoreactivity (IIR) was determined using the Mercodia Iso‐Insulin ELISA, which was demonstrated previously to detect human insulin and cross‐react with most insulin analogues.^24^ PEG precipitation studies incorporated the Iso‐Insulin using a method based on that previously published.^21^ Correlation coefficient was determined using the Pearson R^2^ method. GFC of plasma with and without preincubation of plasma with exogenous human insulin was undertaken as previously described^21^: insulin measurement of GFC fractions using the DiaSorin LIAISON XL chemiluminescent immunoassay, which detects human insulin with low cross‐reactivity with insulin analogues.^24^, ^25^ C‐peptide determinations were made using the LIAISON XL.

## 3 RESULTS

A total of 14 men and 26 women participants with insulin‐treated diabetes (34 designated type 1 and 6 type 2) and labile glycaemic control were evaluated (Table 1; Tables S1 and S2). Serum anti‐insulin IgG was determined for all participants and results ranged from <0.02 to 275 mg/L (Table S3), with 17 within the 0–5 mg/L reference interval (RI).^22^ IIR was also measured in non‐fasting plasma for all participants using the Iso‐Insulin. In 8 participants extremely high apparent concentrations were found, ranging from 7900 pmol/L to around 135,000 pmol/L. IIR in plasma supernatant following PEG precipitation was also determined in all participants, and in 10 controls not treated with insulin. The 95% RI for the ratio of IIR post/pre‐PEG precipitation (PEG%) was 91%–202% for the endogenous insulin present in control plasma across the assay range (data not shown). The apparent increase in IIR following PEG precipitation observed in some subjects is likely due to matrix/calibration effects, as the assay is calibrated against insulin standards in a medium not subjected to PEG precipitation. PEG% was <1% to 176% in the participant group.

**TABLE 1:** Clinical characteristics and medication at presentation of participants with high likelihood of clinically significant anti‐insulin antibodies on initial screen.

Case	Age, year	Sex	Diabetes type	BMI, Kg/m^2^	Other history	Current insulin therapy	Other medications	Presenting Features
1	58	M	T1DM	30.6	Ischaemic heart disease, hypertension, eczema	Porcine (CSII)	Simvastatin, aspirin, candesartan, sitagliptin, GTN	Insulin resistance
2	52	F	T1DM	25.2	Hypertension, depression	25/75 Soluble/protamine insulin lispro	Lisinopril, mirtazapine	Recurrent unpredictable hypoglycaemia
3	56	F	T2DM	22.4	Allogenic BMT for ALL, diabetic nephropathy, left ventricular failure	Glargine U‐200 Lispro U‐200	Candesartan, carvedilol, Fe fumarate, simvastatin, furosemide	Severe insulin resistance (subcutaneous up to 4 units/kg; Intravenous up to 6 units/h).
4	8	F	T1DM	20.4	‐	Aspart (CSII)	‐	Severe nocturnal hypoglycaemia off insulin
5	74	M	T1DM	25.2	Hypothyroidism, pulmonary fibrosis, carotid and coronary artery bypass, ocular thrombosis	Aspart (CSII)	Aspirin, levothyroxine, ramipril, pravastatin, omeprazole, ambulatory oxygen	Recurrent severe nocturnal hypoglycaemia, hypoglycaemia despite suspending insulin therapy
6	64	F	T2DM	24.9	Renal impairment, previous treatment with prednisolone and MMF	Porcine isophane Porcine neutral	Amlodipine, gliclazide, paroxetine, procyclidine, ramipril, risperidone	Morning hypoglycaemia; daytime hyperglycaemia
7	37	M	T1DM	26.1	Ulcerative colitis, Graves' disease, AF, sagittal sinus thrombosis, cerebral haemorrhage, epilepsy	Glargine Glulisine	Carbimazole, adalimumab, aspirin, atorvastatin, escitalopram, metoprolol, lansoprazole, rivaroxaban, Na valproate	Exogenous insulin resistance, recurrent DKA
8	22	F	T1DM	19.5	‐	Detemir Aspart	‐	Insulin resistance

Abbreviations: ALL, acute lymphoblastic leukaemia; BMI, body mass index; BMT, bone marrow transplantation; CSII, continuous subcutaneous insulin infusion; DKA, diabetic ketoacidosis; GTN, glyceryl trinitrate; MMF, mycophenolate mofetil; T1/2DM, Type 1/2 diabetes mellitus.

**FIGURE 1:**
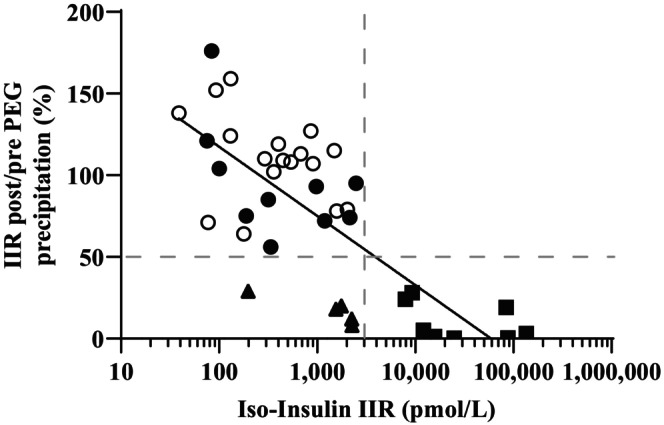
Ratio of insulin immunoreactivity (IIR) post/pre‐PEG precipitation (PEG%) against baseline plasma IIR determined by broad‐specificity immunoassay (‘Iso‐Insulin’) plotted on a log_10_ scale. Correlation of PEG% and Iso‐Insulin IIR using a semilog fit line showed a slope of −40.6 (95% confidence interval −52.7 to −28.6) with a Pearson R^2^ of 0.55. Shaded icons denote anti‐insulin IgG concentrations above upper reference limit, and unshaded icons denote anti‐insulin IgG concentrations within the reference interval. Dotted lines are arbitrary and divide participants into groups deemed to have low probability of clinically significant anti‐insulin antibodies (Group 1: ● or ○), intermediate probability (Group 2: ▲), or high probability (Group 3: ■). 95% reference limits for recovery of endogenous insulin control plasma were 91%–202% at concentrations across the assay range (data not shown).

On plotting PEG% against IIR, three clusters could be discerned (Figure 1). These could be demarcated by thresholds of IIR of 3000 pmol/L and a PEG% of greater than 50%. While these are arbitrary, a plasma insulin concentration of 3000 pmol/L is usually seen only in syndromes of extreme insulin resistance. Indeed we have previously proposed an operational threshold of 1500 pmol/L for endogenous insulin to denote severe insulin resistance after oral glucose challenge in people with a body mass index of below 30 kg/m^2^.^26^ 27 participants had IIR below 3000 pmol/L and a PEG% of above 50%. 10 of this group had an anti‐insulin antibody concentration (IA) above the upper reference limit but based on these biochemical screening assays the probability of pharmacokinetically significant anti‐insulin antibodies in this group was deemed low and no further action was taken. A second group of 5 people also had IIR below 3000 pmol/L, but in this group PEG% was below 50%. All in this group had an (IA) above the upper reference limit, but the probability that these were clinically significant was deemed intermediate. In the final group of 8 participants IIR was above 3000 pmol/L and PEG% was below 50%. All participants in this group had an (IA) above the upper reference limit. These participants were deemed likely to have significant circulating anti‐insulin antibodies based on this initial screen. Plasma from all but one participant in the last two groups was evaluated further using GFC, the ‘gold standard’ assay for circulating insulin antibody complexes. Only participant 9 in the intermediate group was not studied further. They had presented with recurrent ketoacidosis and suddenly reduced insulin requirements, had low IIR of 198 pmol/L with PEG % of 29%, and anti‐insulin IgG concentration of 6 mg/L (RI 0–5), and on this basis were reassigned to the low probability group.

GFC after insulin incubation of plasma from participants 10, 11, 12 and 13 in the intermediate probability group (Figure S1a–d) demonstrated predominantly monomeric IIR in 10, 11 and 12, and predominantly high molecular weight (HMW) IIR in 13, though a HMW fraction was identifiable in all four. This was visible before exogenous insulin incubation only for 11 and 13. Even for 13, however, the amount of antibody‐bound insulin detected was considered to be of uncertain clinical significance. Although some derangement of insulin kinetics could not be excluded in this group, the lack of evidence for immunoglobulins with a high capacity to bind insulin meant that there was insufficient biochemical evidence to support immunodepletion to improve glycaemic control.

**FIGURE 2:**
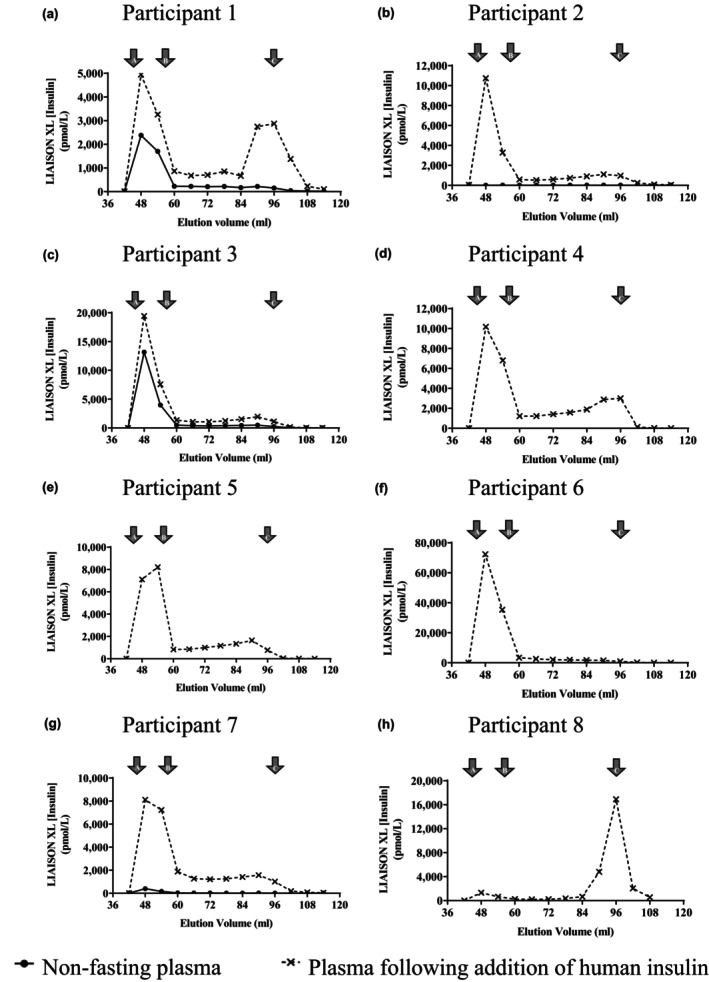
Gel filtration chromatography of participant plasma with and without prior incubation with human insulin. Results of insulin assay after GFC of non‐fasting plasma. Elution volumes of immunoglobulin (a), albumin (b) and monomeric insulin (c) are shown. Insulin concentrations were measured using the DiaSorin LIAISON XL. Results are shown from plasma at presentation, pre‐ and post‐insulin addition (insulin concentrations 13,080 pmol/L and 27,300 pmol/L, respectively) from participant 1 (a); pre‐ and post‐insulin addition (insulin concentrations <3 pmol/L and 27,340 pmol/L, respectively) from participant 2 (b); from plasma at presentation pre‐ and post‐insulin addition (insulin concentrations 35,760 pmol/L and 76,300 pmol/L, respectively) from participant 3 (c); from plasma at presentation post‐insulin addition (insulin concentrations <3 pmol/L pre‐insulin addition and 67,300 pmol/L post‐insulin addition) from participant 4 (d); from plasma at presentation post‐insulin addition (insulin concentrations <3 pmol/L pre‐insulin addition and 49,510 pmol/L post‐insulin addition) from participant 5 (e); from plasma at presentation (insulin concentrations 161,300 pmol/L) from participant 6 (f); from plasma at presentation pre‐ and post‐insulin addition (insulin concentrations 111 pmol/L and 28,660 pmol/L, respectively) from participant 7 (g) and from plasma at presentation post‐insulin addition (insulin concentrations 111 pmol/L pre‐insulin addition and 63,600 pmol/L post‐insulin addition) from participant 8 (h).

In contrast, 7 of the 8 participants in the predefined high probability group showed evidence of HMW IIR on GFC (Figure 2). In 5 cases this was unmasked by preincubation of plasma with soluble human insulin. Based on experience of anti‐insulin antibodies in exogenous insulin‐naïve participants^21^, ^22^ this group were thought to have circulating anti‐insulin antibodies of sufficient concentration and affinity to disturb insulin kinetics, potentially explaining or contributing to labile glycaemic control. This group is discussed further below.

### 3.1 Participant 1

**FIGURE 3:**
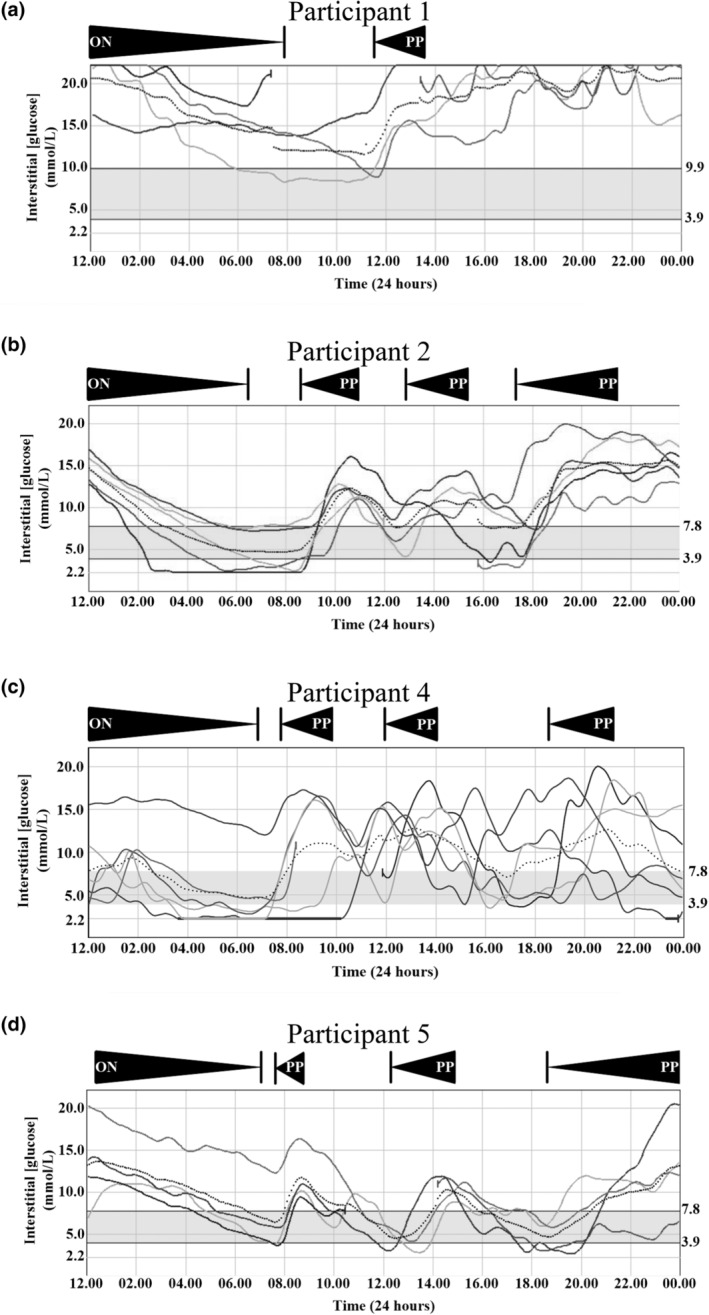
Variable patterns of dysglycaemia at presentation of participants 1 (a), 2 (b), 4 (c), and 5 (d). Demonstration of overnight decrease (ON) and high daytime/postprandial increase (PP) in interstitial blood glucose concentration.

A 58‐year‐old man with a 56‐year history of type 1 diabetes (T1DM) was referred. For 12 years glycaemia had been labile despite meticulous glucose monitoring, structured self management training, and 10 years of continuous subcutaneous insulin infusion (CSII). After trials of insulins including glulisine, aspart and glargine, porcine insulin was currently being used. He had diabetic retinopathy and neuropathy, peripheral vascular disease, and ischaemic heart disease. Intractably poor glycaemic control led to assessment for pancreatic islet transplantation; however, an atypical glycaemic profile and detection of anti‐insulin antibodies prompted further evaluation. His glycaemic profile showed sustained hyperglycaemia, worse postprandially, with concentration falling overnight (Figure 3a). Insulin had severely delayed glucose‐lowering action, and varying short acting doses by up to 20 units failed to improve control. Regular oral carbohydrate was required to avoid hypoglycaemia. Repeated attempts to follow dose adjustment advice had resulted in dysglycaemia, engendering severe anxiety about hypoglycaemia.

IIR (non‐fasting plasma) suggested extreme hyperinsulinaemia, PEG% was low at 1%, and anti‐insulin antibodies were strongly detected (Tables S3 and S4). HMW IIR due to insulin antibody complex formation was confirmed by GFC. HMW IIR increased after preincubation of plasma with exogenous soluble human insulin, consistent with insulin binding by antibody, confirming the presence of antibody with a high capacity to bind insulin reversibly (Figure 2a).

Given strong biochemical evidence for an anti‐insulin antibody as the cause of labile glycaemia, immunomodulatory therapy was introduced. Initial treatment with mycophenolate mofetil and then rituximab yielded neither clinical not biochemical improvement, so to provide evidence that antibody depletion could be effective plasmapheresis was undertaken. This convincingly reduced (IA) and IIR, abolishing the magnitude of IIR increase formerly seen in dilution studies, and increased PEG% into the reference interval. Despite this biochemical evidence of pathogenic antibody depletion, little clinically meaningful improvement in glycaemia was discerned. HbA_1c_ was unchanged, likely because anxiety about hypoglycaemia proved a barrier to adequate insulin dose titration. Follow up with pulsed intravenous cyclophosphamide was stopped after 3 months due to recurrent neutropenia. Three months later abundant HMW insulin was again found on GFC (data not shown), with a biochemical profile resembling baseline (Table S4). Renal function continued to deteriorate requiring thrice weekly haemodialysis, and cardiovascular fitness for kidney‐pancreas transplantation is currently being evaluated.

### 3.2 Participant 2

A 52‐year‐old woman with a 43‐year history of T1DM was referred with daytime/postprandial hyperglycaemia and nocturnal hypoglycaemia (Figure 3b). She reported unpredictability of insulin action first noticed several years after diagnosis. Labile glycaemia was sufficiently disabling to preclude full time work. Hypoglycaemia awareness was reduced, but no microvascular complications of diabetes were documented. CSII was declined, and many different insulin regimens, encompassing different insulin preparations and dosing schedules, had failed to improve glycaemic control resulting in clinical suspicion of wilful insulin maladministration. At referral she was treated with once daily biphasic analogue insulin lispro (32 units/day of 25% lispro, 75% lispro protamine suspension). Grossly elevated IIR was revealed by immunoassay of non‐fasting plasma, with PEG% of 5% (Table S3). IAs were strongly detectable, and GFC demonstrated HMW IIR after preincubation of plasma with native insulin (Figure 2b). The participant declined immunomodulatory therapy after consideration of its risks and benefits, and instead has used a continuous glucose monitoring (CGM) system, which she describes as life changing, continuing once daily biphasic insulin, injecting this up to 1.5 h before breakfast to try and minimise postprandial hyperglycaemia. CGM alarms have helped pre‐empt hypoglycaemia, but weight has increased by 10 kg despite a reduction of insulin dose to 24 units/day. She has reported benefit from having the cause of her labile glycaemia explained, alleviating anxiety that she was ‘to blame’.

### 3.3 Participant 3

A 56‐year‐old lean woman (BMI 21.1 kg/m^2^) with a 14‐year history of diabetes was referred with poor glycaemic control. After good control for 12 years on metformin and gliclazide, secondary deterioration, with HbA_1c_ rising from 57 to 115 mmol/mol (7.4% to 12.7%), had led to starting of subcutaneous insulin. She had a history of acute lymphoblastic leukaemia cured by allogenic bone marrow transplant 21 years earlier, diabetic nephropathy (eGFR 26 mL/min), and severe left ventricular dysfunction. Despite insulin doses up to 4 units/kg/day, hyperglycaemia had persisted. Biphasic soluble and isophane recombinant human insulin, or biphasic soluble and protamine insulin lispro had been tried without improvement, while inpatient evaluation revealed hyperglycaemia refractory to intravenous insulin at up to 6 units/hour while fasting. The finding of anti‐insulin antibodies at 275 mg/L prompted referral for further evaluation. At this point she was taking U300 glargine and U200 lispro, at a total daily dose of around 4 units/kg.

Strongly positive IAs were confirmed, and high IIR was shown in non‐fasting plasma by broad‐specificity immunoassay, with PEG% of <1% (Table S3). GFC with insulin preincubation again demonstrated marked HMW IIR, both with and without preincubation of plasma with human insulin (Figure 2c). Immunomodulatory therapy was considered but decided against due to concern about cardiovascular risk. Glycaemia gradually improved with switching to more concentrated insulins, continued insulin dose titration and introduction first of linagliptin and later liraglutide despite low BMI. This was based on evidence that cytotoxic treatments and whole body irradiation may damage adipose tissue, producing insulin resistance and a ‘metabolically obese’ profile even at low body mass index.^27^ HbA_1c_ settled to 70–80 mmol/mol (8.6%–9.5%) on a total daily insulin dose of 3.5 units/kg/day and 1.2 mg liraglutide daily. This was achieved without hypoglycaemia, but with continued marked postprandial hyperglycaemia despite 30 units of preprandial lispro.

### 3.4 Participant 4

An 8‐year‐old girl with a 5‐year history of T1DM presented with unpredictable hypoglycaemia and increasing HbA_1c_. Treatment had been with insulin aspart via CSII since 4 years old. At 6 years old an atypical profile of basal to bolus insulin was noted, with only 3% of insulin delivered being basal. By 8 years old glucose concentrations began dropping overnight despite interruption of basal insulin infusion, while spikes of hyperglycaemia were observed postprandially (Figure 3c), and HbA_1c_ rose to 68 mmol/mol (8.4%). Her parent would aim for a bedtime blood glucose of around 15 mmol/L to minimise hypoglycaemia risk overnight. She was admitted for serial biochemical evaluation during supervised subcutaneous insulin therapy. Strongly positive anti‐GAD and anti‐IA2 antibodies were confirmed, and blood glucose was confirmed to fall overnight despite lack of exogenous insulin. C‐peptide was undetectable, but plasma IIR was grossly elevated to >20,000 pmol/L throughout the night.

On specialised evaluation IAs were strongly detected, with reduced PEG% (19%) (Table S3). GFC was consistent with the presence of antibody with a high capacity to bind insulin, unmasked by preincubation of plasma with recombinant human insulin (Figure 2d). A range of therapeutic options including immunomodulatory therapy were considered. In view of improving HbA_1c_, absence of moderate/severe hypoglycaemia or ketosis and severe procedural anxiety, a conservative approach was adopted including parental explanation and support. A 4‐month trial of changing CSII insulin to lispro was ineffective, and insulin aspart was recommenced at the parent's request. The CSII system was upgraded to a Tandem T slim™ device with Basal IQ^tm^ supported by a Dexcom G6™ CGMS with hypoglycaemia alarm.

HbA_1c_ continued to improve to 41 mmol/mol (5.9%), with CGMS showing 72% time in range with low hypoglycaemia risk. At last review the pattern of insulin delivery had not changed significantly but the CGMS system had allowed greater parental confidence and improved management. Despite this, a significant amount of parental input is still required to manage glycaemia safely, especially overnight. To avoid postprandial spikes, eating is often delayed by up to 90 minutes after an insulin bolus. Psychological therapy for procedural anxiety permitted repeat investigations, which showed continued presence of antibodies with a high insulin‐binding capacity and no discernible change from initial evaluation.

### 3.5 Participant 5

A 74‐year‐old man with T1DM for 68 years was referred with labile glycaemia for 10 years, characterised by daytime hyperglycaemia and severe matutinal hypoglycaemia confirmed by continuous glucose monitoring (Figure 3d). He had severely blunted hypoglycaemia awareness. Over 15 years, insulin had been administered by CSII, lately with insulin aspart. Hypoglycaemia occurred despite stopping insulin from 20.00 h to 07.00 h and consuming a snack (~50 g carbohydrate) before bed. Unsuccessful alternative treatment approaches had included basal‐bolus insulin regimes using a variety of insulins and insulin analogues including recombinant human insulin, lispro, and detemir.

High IIR was confirmed by immunoassay of non‐fasting plasma with a PEG% of 28%, (Table S3). IAs were strongly detectable, and GFC with insulin preincubation again demonstrated an increase in HMW IIR, suggesting the presence of an antibody with a high capacity to bind insulin (Figure 2e).

A trial of prednisolone was ineffective. A Medtronic 670G Pump was introduced, and nocturnal hypoglycaemia was improved with introduction of a 40–60 g complex carbohydrate snack before bed. Hypoglycaemia towards lunchtime was combated by pre‐emptive short‐term suspension of basal insulin. With these conservative measures average glucose concentration recorded by a Freestyle® Libre monitor was 10 mmol/L with a glucose management indicator of 7.6% and glucose variability of 29%. 50% of readings were within target range, 49% above and 1% below. Plasmapheresis and immunosuppressive therapy have been discussed but are being reserved as a contingency option.

### 3.6 Participant 6

A 64‐year‐old man with type 2 diabetes was referred with recurrent daytime hyperglycaemia and morning hypoglycaemia. He had a history of cutaneous hypersensitivity to subcutaneous administration of a range of insulin analogues and had settled on basal‐bolus porcine insulin as the best tolerated, at total daily doses of around 340 units. Anti‐insulin antibodies had been detected and treatment with prednisolone and mycophenolate mofetil previously tried with no benefit.

High IIR was confirmed by immunoassay of non‐fasting plasma, with PEG% of 3% (Table S3). IAs were strongly detectable, and GFC with insulin preincubation demonstrated HMW IIR (Figure 2f). 4 doses of rituximab were given following which total daily insulin dose reduction was possible from 340 units to 150 units, with resolution of hypoglycaemia.

### 3.7 Participant 7

A 37‐year‐old man with a 5‐year history of T1DM treated with insulins glulisine and glargine was evaluated due to labile glycaemia and recurrent diabetic ketoacidosis (DKA). HbA_1c_ was 107 mmol/mol (11.9%) despite 4 units/kg/day insulin. C‐peptide was undetectable and there were no other clinical features of insulin resistance.

High IIR was confirmed by immunoassay of non‐fasting hyperglycaemic plasma, which had PEG% of <1% (Table S3). IAs were strongly detectable, and GFC with insulin preincubation again demonstrated increased HMW IIR (Figure 2g). FreeStyle® Libre monitoring revealed asymptomatic postprandial hypoglycaemia. Insulin therapy was changed to insulins detemir and lispro (total daily dose 3–4 units/kg) with subsequent reduction in HbA_1c_ to 74 mmol/mol (8.9%). Immunosuppression was considered impractical in the face of neurological disability secondary to previous intracerebral haemorrhage.

### 3.8 Participant 8

A 22‐year‐old woman with T1DM presented with hyperglycaemia and insulin resistance, (1 unit insulin aspart per 4 g carbohydrate and 80 units insulin detemir daily) interspersed with unpredictable hypoglycaemia. She also experienced erythema and pruritis at insulin injection sites. Anti‐human insulin IgE was not detected, and anti‐insulin IgG was 7 (0–5) mg/L. IIR was 7900 pmol/L with PEG% of 24%. GFC with insulin preincubation identified a very small amount of insulin antibody complexes (Figure 2h) and these were deemed insufficient to explain glycaemic instability. A trial of CSII was planned at the time of writing.

**FIGURE 4:**
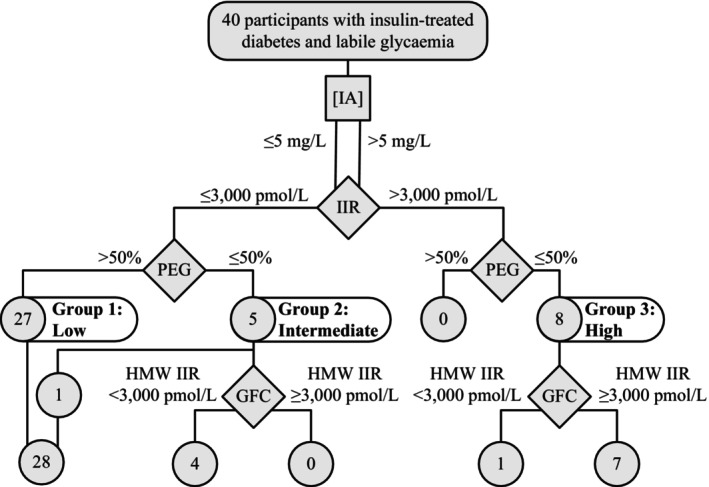
Algorithm used for biochemical evaluation of participants with clinically suspected anti‐insulin antibodies. Gel filtration chromatography (GFC); high molecular weight insulin immunoreactivity (HMW IIR); anti‐insulin IgG measurement ([IA]); plasma insulin immunoreactivity (IIR); IIR recovery in supernatant following PEG precipitation and centrifugation of plasma (PEG). Positive GFC results determined if HMW immunoreactivity >3000 pmol/L post‐insulin spike. One participant in Group 2 did not have GFC and was not studied further.

An investigation algorithm and summary of biochemical findings for the cohort studied is presented in Figure 4. This demonstrates that GFC in the participants studied confirmed large amounts of circulating insulin‐binding capacity in all but one of the participants with high IIR and low PEG%, with only small levels detected in the group with IIR below 3000 pmol/L and low PEG%. This demonstrates that IIR and PEG precipitation were sufficient in this series to discriminate most participants with high levels of insulin‐binding capacity in the plasma. GFC refined and confirmed this, while also identifying lower levels of insulin binding in other groups.

## 4 DISCUSSION

Unstable glycaemia in insulin‐treated diabetes usually relates to suboptimal insulin management skills, psychological factors, and/or variable insulin absorption. Perturbation of insulin pharmacokinetics by high capacity anti‐insulin antibodies is an important differential diagnosis, yet poor awareness, and lack of clarity about optimal laboratory work up commonly delay or prevent diagnosis. An insulin ‘neutralising’ factor associated with insulin administration was first reported in 1938,^28^ with anti‐insulin antibodies demonstrated in 1955.^1^ It was hypothesised that such antibodies may underlie ‘brittle diabetes’, although the potential of antibodies to enhance glucose control by sustaining insulin action was also noted.^29^, ^30^

We recently described an approach to detecting significant anti‐insulin antibodies in exogenous insulin‐naïve participants, and now adapt this to insulin‐treated diabetes. Immunoassay‐based detection of antibodies is a useful screen but does not establish clinical significance. Assays are moreover non‐standardised, will not detect all antibody classes, and have specificity for human insulin. Antibody concentrations are best interpreted with plasma insulin concentration, but insulin analogue cross‐reactivity in immunoassays is variable^24^, ^25^ and antibodies may interfere.^21^ Despite these limitations, we demonstrate that a well characterised broad‐specificity insulin immunoassay is informative, though values yielded will not faithfully reflect true plasma insulin concentration, with implausibly high immunoreactivity being a clue to significant anti‐insulin antibodies.

PEG precipitation increases diagnostic confidence, although knowledge of assay performance in this context is required. Particular caution applies to insulin analogues incorporating fatty acid moieties (e.g. insulins detemir, degludec), which may produce high IIR with low PEG% independent of antibodies (in‐house observations). PEG precipitation is moreover not reliable for insulin‐binding IgA.^22^ GFC with insulin preincubation is definitive in both situations. Despite these caveats, we report that an easily implementable combination of antibody detection, broad‐specificity insulin immunoassay, and PEG precipitation identified all participants in our series with circulating insulin antibody complexes, as identified by GFC. Sensitivity of GFC was enhanced through preincubation of plasma with soluble human insulin, unmasking HMW insulin in 5 of 7 positive cases.

The 7 participants we identified as having plausibly clinically significant antibodies on biochemical grounds all showed postprandial hyperglycaemia, with falling glucose concentrations overnight, often leading to hypoglycaemia. This is predicted by the delayed clearance of insulin by anti‐insulin antibodies, which effectively convert rapid acting insulin into a long‐acting preparation. In two participants, high insulin requirements were seen, although in one of these prior treatment for acute lymphoblastic leukaemia was an alternative explanation.^27^ The primary benefit of detecting clinically significant anti‐insulin antibodies in this study was to offer an explanation to participants, families and caregivers of the atypical glycaemic profile, allowing rational adjustment of insulin regimens while alleviating suspicion of surreptitious dose manipulation.

Improved glycaemic control on antibody depletion is the ultimate proof of the clinical significance of these antibodies. This has been reported in some previous insulin‐treated cases using a variety of immunosuppressive regimens,^10^, ^13^, ^17^, ^20^ and in exogenous insulin‐naïve participants with IAS, where the effect of antibodies on insulin kinetics are easier to discern.^22^, ^23^ However, benefit is not universally seen, and reporting bias in favour of positive results is likely. Moreover, depletion of antibodies may be ineffective or transient in the presence of long‐lived plasma cells, is logistically burdensome for participants, and runs the risk of iatrogenic complications such as opportunistic infection and side effects of high dose glucocorticoids, depending on the regimen chosen.

In our published series,^22^ participant 6 appeared to benefit from immunodepletion, but participant 1 received repeated antibody‐depleting therapies with little beneficial effect, despite convincing biochemical evidence of high plasma insulin‐binding capacity and short‐term improvement in glycaemia acutely following plasma exchange, and several iatrogenic complications accrued. This emphasises the complex risk–benefit evaluation in considering immunomodulatory therapy to deplete anti‐insulin antibodies. A particular challenge lies in identifying a reliable index of efficacy, especially where, as in participant 1 in this report, entrenched habits and fears acquired over a long diabetes course made it difficult to take advantage of antibody depletion.

It is a possibility that anti‐insulin antibodies may also have perturbed insulin pharmacokinetics in the biochemically intermediate group; however, this is unproven and requires further study. A study comparing antibody prevalence and characterisation in participants with labile glycaemia despite intensive insulin management and those with excellent control may be useful in this context. In the interim, we suggest that our pragmatic approach to detecting likely significant anti‐insulin antibodies is applicable in current practice and should be considered in any insulin‐treated participant with unexplained labile glycaemia, particularly where overnight hypoglycaemia and postprandial hyperglycaemia are prominent.

## FUNDING INFORMATION

D.C. was funded by the Diabetes Research & Wellness Foundation (Sutherland–Earl Clinical Fellowship) and R.K.S. by the Wellcome Trust (grant 210752/Z/18/Z). Funding was also received from the Medical Research Council (MRC_MC_UU_12012/5) and the United Kingdom National Institute for Health Research (NIHR) Cambridge Biomedical Research Centre (to D.C.).

## CONFLICT OF INTEREST STATEMENT

The authors declare that they have no conflicts of interest. R.K.S. is the guarantor of the study.

## Supporting information


Figure S1.

Table S1.
Table S2.
Table S3.
Table S4.

## Data Availability

All relevant data are presented.
