# Supplementary material for: Diagnosis and treatment of anti‐insulin antibody‐mediated labile glycaemia in insulin‐treated diabetes
Source: Diabet Med. 2023 Sep 1;40(11):e15194. doi: 10.1111/dme.15194 (PMC10946589; doi:10.1111/dme.15194)
Supplement: Supplementary file 1 — Figure S1. [file DME-40-0-s002.pptx]

## Slide 1
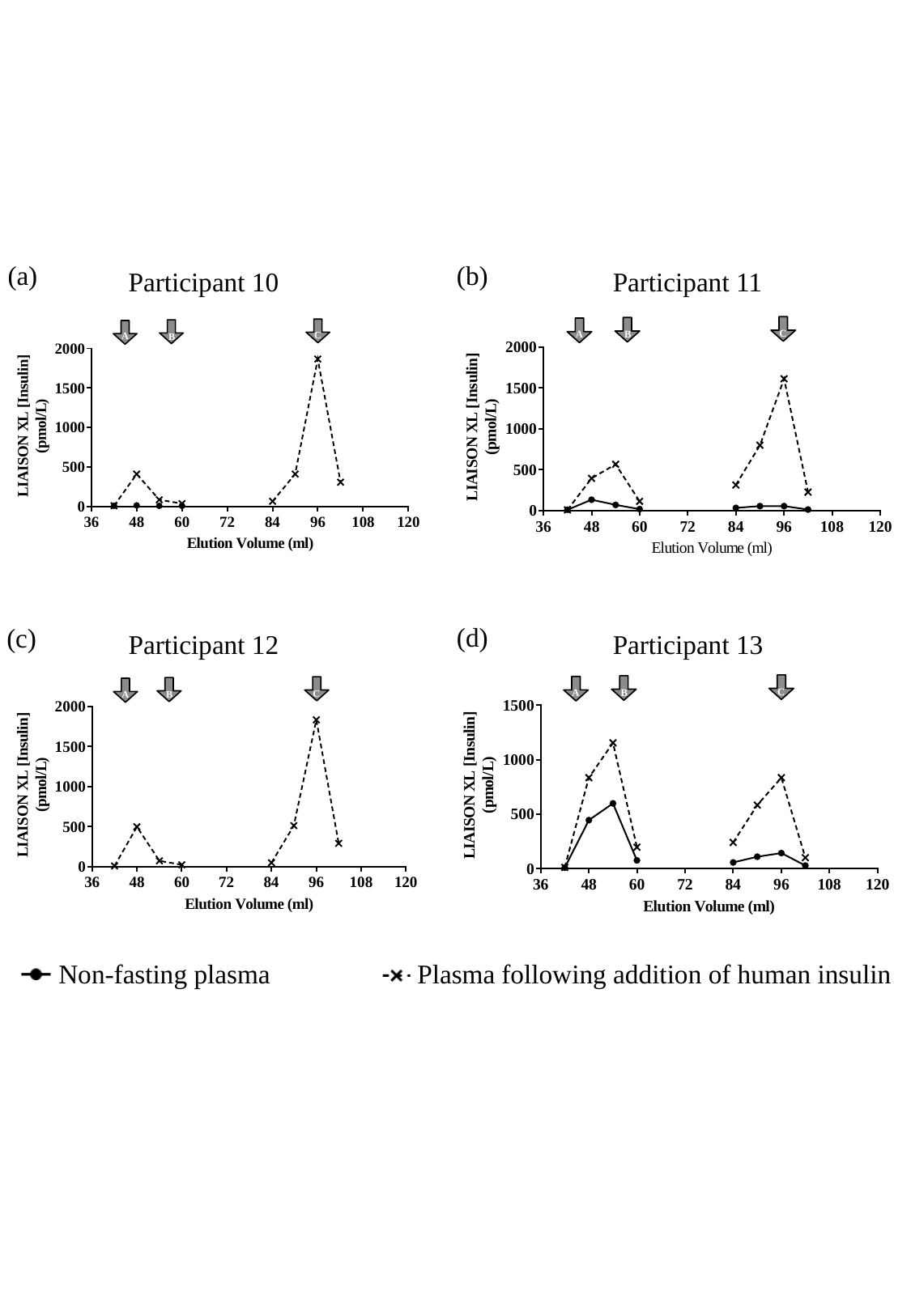

(b)
(a)
Participant 10
Participant 11
(d)
(c)
Participant 12
Participant 13
Plasma following addition of human insulin
Non-fasting plasma
