# Supplementary material for: Diagnosis and treatment of anti‐insulin antibody‐mediated labile glycaemia in insulin‐treated diabetes
Source: Diabet Med. 2023 Sep 1;40(11):e15194. doi: 10.1111/dme.15194 (PMC10946589; doi:10.1111/dme.15194)
Supplement: Supplementary file 2 — Table S1. Table S2. Table S3. Table S4. [file DME-40-0-s001.docx]

**Supplementary Online Data**

**Diagnosis and treatment of anti-insulin antibody-mediated labile glycaemia in insulin-treated diabetes**

David S Church^1,2^, Peter Barker^3^, Keith A. Burling^3^, Shah K. Shinwari^4^, Carmel Kennedy^5^, Diarmuid Smith^5^, David P. Macfarlane^6^, Andrew Kernohan^7^, Anna Stears^8^, Muhammad A. Karamat^4^, Karen Whyte^9^, Parth Narendran^10^, David J. Halsall^1^, Robert K. Semple^11,2^

^1^Department of Clinical Biochemistry and Immunology, Cambridge University Hospitals NHS Foundation Trust, Cambridge CB2 0QQ

^2^The University of Cambridge MRC Metabolic Disease Unit, Wellcome Trust-MRC Institute of Metabolic Science, Cambridge, CB2 0QQ, United Kingdom

^3^Core Biochemical Assay Laboratory, NIHR Cambridge Biomedical Research Centre, Cambridge CB2 0QQ, United Kingdom

^4^Diabetes & Endocrinology Centre, Birmingham Heartlands Hospital, Birmingham B9 5SS

^5^Department of Diabetes and Endocrinology, Beaumont Hospital, RCSI Medical School Dublin

^6^Department of Diabetes & Endocrinology, Raigmore Hospital, Inverness IV2 3UJ, United Kingdom

^7^Department of Diabetes and Endocrinology, Queen Elizabeth University Hospital, Glasgow G51 4TF

^8^National Severe Insulin Resistance Service, Wolfson Diabetes & Endocrine Clinic, Cambridge University Hospitals NHS Foundation Trust, Cambridge CB2 0QQ

^9^West Glasgow Ambulatory Care Hospital, Glasgow G3 8SJ

^10^Institute of Metabolism and Systems Research, College of Medical and Dental Sciences, University of Birmingham, Edgbaston B15 2TT

^11^University of Edinburgh Centre for Cardiovascular Science, Queen’s Medical Research Institute, Edinburgh EH16 4TJ, United Kingdom

| **Case** | **Age, yr** | **Sex** | **Diabetes type** | **B.M.I.**  **Kg/m^2^** | **Other history** | **Current insulin therapy** | **Other medication** | **Presenting Features** |
| --- | --- | --- | --- | --- | --- | --- | --- | --- |
| **9** | 43 | F | T1DM | 27.8 | diabetic gastroparesis, stroke | glargine, aspart (CSII) | atorvastatin, clopidogrel, codeine, lansoprazole, paracetamol, pregabalin, quinine sulphate | recurrent DKA, previous insulin resistance, recent marked reduction of insulin requirement |
| **10** | 16 | F | T1DM | 25.4 | migraine, chronic back pain | 30/70 soluble/ protamine insulin, lispro, aspart | - | insulin resistance,  severe cutaneous insulin hypersensitivity |
| **11** | 47 | F | T1DM | 28.0 | long QT syndrome | human isophane insulin, lispro | metformin, omeprazole, simvastatin | severe hyperglycaemia, unpredictable hypoglycaemia. |
| **12** | 29 | F | T1DM | 21.4 | coeliac disease | detemir, aspart | - | variable insulin sensitivity |
| **13** | 45 | F | T2DM | 27.0 | diabetic neuropathy, hypertension | human isophane insulin | dapagliflozin, exenatide, ramipril, simvastatin, zopiclone, pregabalin, sertraline, pramipexole, beclomethasone, salbutamol, Li carbonate | insulin resistance, hypoglycaemia |

**Supplementary Table 1: Clinical characteristics and medication at presentation of patients with intermediate likelihood of clinically significant anti-insulin antibodies on initial screen;** B.M.I. = body mass index; T1/2DM = Type 1/2 diabetes mellitus; CSII – continuous subcutaneous insulin infusion; DKA = diabetic ketoacidosis

| **Case** | **Age, y** | **Sex** | **Diabetes type** | **B.M.I.**  **Kg/m^2^** | **Other history** | **Current insulin therapy** | **Other medication** | **Presenting Features** |
| --- | --- | --- | --- | --- | --- | --- | --- | --- |
| **14** | 44 | F | T1DM | N/A | myasthenia gravis, vitiligo, Budd-Chiari syndrome, thrombophilia | 25/75 soluble/ protamine human insulin | gliclazide, metformin, lactulose, warfarin, cetirizine | cutaneous reaction,  unpredictable insulin action |
| **15** | 16 | F | T1DM | N/A | - | glargine, apidra | metformin | insulin resistance |
| **16** | 29 | M | T1DM | 31.3 | hypertension | human insulin, aspart | acarbose, topiramate, escitalopram, lisinopril, oxycodone | recurrent hypoglycaemia, hyperglycaemia |
| **17** | 44 | F | T1DM | 33.3 | lumbar spondylosis, hysterectomy, deep vein thrombosis, hypothyroidism | detemir, aspart | sitagliptin, levothyroxine, simvastatin, spironolactone, tramadol | recurrent severe hypoglycaemia |
| **18** | 17 | F | T1DM | 19.0 | - | aspart (CSII) | - | recurrent hypoglycaemia |
| **19** | 56 | M | T2DM | 39.6 | - | detemir, lispro | metformin, simvastatin | morning hypoglycaemia, protracted insulin action |
| **20** | 34 | M | T2DM | 27.0 | - | degludec, glulisine | gabapentin, atorvastatin | recurrent nocturnal hypoglycaemia |
| **21** | 12 | F | T1DM | 17.9 | - | glargine, glulisine | metformin | labile glycaemia,  recurrent hypoglycaemia |
| **22** | 23 | M | T1DM | 26.3 | - | glargine, lispro | - | recurrent hypoglycaemia despite low insulin dose |
| **23** | 32 | M | T1DM | 23.3 | - | glargine, aspart | - | recurrent prolonged hypoglycaemia |
| **24** | 43 | M | T1DM | 27.4 | - | detemir, lispro | dapagliflozin, atorvastatin | severe insulin resistance |
| **25** | 59 | F | T1DM | 21.9 | gastroparesis, Raynaud’s phenomenon, hypothyroidism | aspart (CSII) | levothyroxine, amitriptyline, ascorbic acid, hydroxychloroquine, lansoprazole, metoclopramide, simvastatin | labile glycaemia |
| **26** | 45 | F | T1DM | 25.4 | - | human insulin  U-500 | liraglutide, metformin, atorvastatin | severe insulin resistance |
| **27** | 17 | F | T1DM | 26.9 | - | degludec  glulisine (CSII) | - | insulin resistance, prolonged hypoglycaemia |
| **28** | 20 | F | T1DM | 24.0 | bilateral band-like lipodystrophy | detemir, aspart | - | recurrent severe nocturnal hypoglycaemia |
| **29** | 67 | F | T1DM | 36.6 | asthma, gastroparesis | detemir, aspart | sertraline, Fe sulphate, amitriptyline, simvastatin, salmeterol | labile glycaemia |
| **30** | 15 | F | T1DM | N/A | - | glargine, aspart | - | subcutaneous insulin resistance |
| **31** | 45 | F | T1DM | N/A | depression | lispro | sertraline | severe recurrent hypoglycaemia |
| **32** | 46 | F | T2DM | 37.2 | asthma, hypertension, peripheral neuropathy | glargine, aspart | aspirin, furosemide, salbutamol, ipratropium, carvedilol, atorvastatin, pregabalin, perindopril, spironolactone, tramadol | subcutaneous insulin resistance |
| **33** | 46 | M | T1DM | 37.6 | sleeve gastrectomy | glargine, glulisine | - | labile glycaemia |
| **34** | 20 | F | T1DM | 27.5 | autonomic neuropathy,  hypothyroidism  pulmonary embolism | lispro (CSII) | levothyroxine, Fe sulphate, enoxaparin | recurrent ketoacidosis, prolonged hypoglycaemia despite no insulin therapy |
| **35** | 74 | M | T1DM | 26.3 | - | hypurin neutral | - | recurrent hypoglycaemia |
| **36** | 9 | M | T1DM | 16.6 | - | human insulin | - | prolonged hyperglycaemia, sudden nocturnal hypoglycaemia |
| **37** | 36 | M | T1DM | 27.3 | hyper-cholesterolaemia | glulisine | atorvastatin | delayed-onset nocturnal hypoglycaemia despite little/no insulin |
| **38** | 54 | F | T1DM | 23.0 | - | detemir, lispro | - | insulin resistance |
| **39** | 55 | M | T1DM | 25.2 | asthma, hypertension | degludec, aspart | atorvastatin, ramipril, aspirin salbutamol | recurrent hypoglycaemia, acute decrease in insulin requirement |
| **40** | 93 | F | T1DM | 24.9 | renal impairment, retinopathy, hypertension peripheral vascular disease, heart failure, meningioma | protamine human insulin, aspart | ferrous fumarate, levetiracetam, doxazosin, lansoprazole, amlodipine, aspirin, enoxaparin, folate | recurrent hypoglycaemia,  unpredictable severe hyperglycaemia |

**Supplementary Table 2: Clinical characteristics and medication at presentation of patients with low likelihood of clinically significant anti-insulin antibodies on initial screen;** B.M.I. = body mass index; CSII – continuous subcutaneous insulin infusion; DKA = diabetic ketoacidosis; GTN = glyceryl trinitrate; T1/2DM = Type 1/2 diabetes mellitus.

| **Likelihood of significant anti-insulin antibodies on initial screen** | **Case** | **Anti-insulin IgG, mg/L**  (0-5) | **Iso-Insulin insulin immunoreactivity, pmol/L**  (12-150) | **Post/pre PEG precipitation, %**  (>91%) | **C-peptide immunoreactivity, pmol/L**  (174-960) |
| --- | --- | --- | --- | --- | --- |
| **High** | **1** | 18 | 15,700 | 1 | 18 |
|  | **2** | 25 | 12,050 | 5 | <9 |
|  | **3** | 132 | 87,800 | <1 | 3230 |
|  | **4** | 19 | 84,328 | 19 | <9 |
|  | **5** | 64 | 9,290 | 28 | <9 |
|  | **6** | 275 | 135,336 | 3 | 3060 |
|  | **7** | 196 | 24,913 | <1 | <9 |
|  | **8** | 7 | 7,900 | 24 | <9 |
| **Intermediate** | **9** | 6 | 198 | 29 | <9 |
|  | **10** | 18 | 1,545 | 18 | 246 |
|  | **11** | 10 | 1,758 | 20 | - |
|  | **12** | 10 | 2,257 | 8 | - |
|  | **13** | 20 | 2,246 | 12 | 894 |
| **Low** | **14** | 9 | 335 | 56 | - |
|  | **15** | 8 | 189 | 75 | - |
|  | **16** | 2 | 77 | 71 | - |
|  | **17** | 15 | 2,153 | 74 | <9 |
|  | **18** | 5 | 39 | 138 | 80 |
|  | **19** | <0.02 | 540 | 108 | - |
|  | **20** | 3 | 860 | 127 | 329 |
|  | **21** | 3 | 906 | 107 | - |
|  | **22** | 3 | 449 | 109 | 30 |
|  | **23** | 3 | 93 | 152 | - |
|  | **24** | 10 | 2,488 | 95 | - |
|  | **25** | 7 | 100 | 104 | - |
|  | **26** | 7 | 318 | 85 | - |
|  | **27** | 2 | 1,578 | 78 | <9 |
|  | **28** | 2 | 403 | 119 | - |
|  | **29** | 6 | 1,192 | 72 | - |
|  | **30** | 6 | 977 | 93 | - |
|  | **31** | 4 | 131 | 159 | - |
|  | **32** | 2 | 361 | 102 | 1140 |
|  | **33** | 13 | 84 | 176 | - |
|  | **34** | 4 | 1,495 | 115 | - |
|  | **35** | 6 | 75 | 121 | 30 |
|  | **36** | 2 | 178 | 64 | <9 |
|  | **37** | 2 | 291 | 110 | 93 |
|  | **38** | 5 | 2,025 | 79 | <9 |
|  | **39** | 3 | 681 | 113 | <9 |
|  | **40** | 4 | 130 | 124 | <9 |

**Supplementary Table 3: Non-fasting blood test results at presentation.**

| **Day from first evaluation** | 0 | 262 | 278 | 369 | 601 | 808 | 822 | 829 | 907 | 965 |
| --- | --- | --- | --- | --- | --- | --- | --- | --- | --- | --- |
| **[Anti-Ins IgG]** | 18 | -- | 14 | 12 | 11 | 15 | 4 | 2 | 13 | 16 |
| **IIR, pmol/L** | 15,700* | -- | 2,169 | 1,633 | 1,415 | 175 | 390 | 121 | 634* | 600 |
| **IIR, pmol/L**  **(1:4)** | -- | -- | -- | 5,980 | 2,934 | 223 | <90 | <90 | 1230 | 1,185 |
| **IIR, pmol/L (1:49)** | -- | -- | -- | -- | 4,542 | <900 | <900 | <900 | 1550 | 1,500 |
| **Post PEG IIR, %** | 1 | 13 | 8 | 7 | 19 | 69 | 190 | -- | 9 | 12 |
| **Immuno-suppression** | -- | MMF | MMF |  | post Rituximab | pre PE | post  7 PE | post 10 PE | pre 5^th^ pulse i/v CyP | -- |

**Supplementary Table 4: Serial biochemical evaluation of patient 1.** *gel filtration chromatography performed at these points demonstrated insulin immunocomplexes; IIR = insulin immunoreactivity (‘Iso-insulin’); PEG = polyethylene glycol; MMF = mycophenolate mofetil; PE = plasma exchange; CyP = cyclophosphamide
